# 1-(4-Chloro­phen­yl)-4,4-dimethyl­pent-1-en-3-one

**DOI:** 10.1107/S1600536809004358

**Published:** 2009-02-28

**Authors:** Tian-Quan Wu, Lin Xia, Ai-Xi Hu, Jiao Ye

**Affiliations:** a College of Chemistry and Chemical Engineering, Hunan University 410082, Changsha, People’s Republic of China

## Abstract

In the title compound, C_13_H_15_ClO, the carbonyl and ethenyl groups are not coplanar with benzene ring system, forming dihedral angles of 35.37 (5) and 36.27 (11)°, respectively. The mol­ecules are packed in an offset face-to-face arrangement showing π–π stacking inter­actions involving the benzene rings [centroid–centroid distance = 3.586 (4) Å].

## Related literature

The title compound is an important inter­mediate in the pesticide industry, see: Wang *et al.* (2006 ▶). For related structures, see: Anuradha *et al.* (2008 ▶); Butcher *et al.* (2007 ▶); Gong *et al.* (2008 ▶); Harrison *et al.* (2007 ▶); Patil *et al.* (2007 ▶); Sarojini *et al.* (2007 ▶); Thiruvalluvar *et al.* (2007 ▶); Thiruvalluvar *et al.* (2008 ▶); Xia & Hu (2008 ▶).
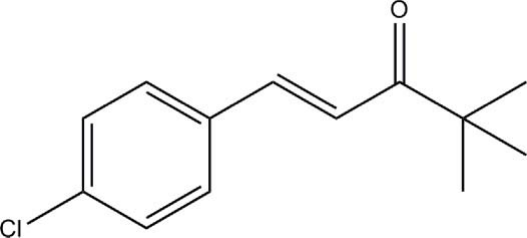


## Experimental

### 

#### Crystal data

C_13_H_15_ClO
*M* *_r_* = 222.70
Triclinic, 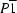

*a* = 5.6831 (4) Å
*b* = 9.9156 (6) Å
*c* = 11.3731 (7) Å
α = 103.487 (1)°
β = 101.160 (1)°
γ = 103.697 (1)°
*V* = 584.12 (7) Å^3^
*Z* = 2
Mo *K*α radiation
μ = 0.30 mm^−1^
*T* = 173 (2) K
0.46 × 0.31 × 0.21 mm

#### Data collection

Bruker SMART 1000 CCD diffractometer
Absorption correction: multi-scan (*SADABS*; Sheldrick, 2004 ▶) *T*_min_ = 0.875, *T*_max_ = 0.940
4545 measured reflections
2251 independent reflections
1989 reflections with *I* > 2σ(*I*)
*R*_int_ = 0.016

#### Refinement

*R*[*F* ^2^ > 2σ(*F* ^2^)] = 0.032
*wR*(*F* ^2^) = 0.094
*S* = 1.07
2251 reflections
139 parameters
H-atom parameters constrained
Δρ_max_ = 0.26 e Å^−3^
Δρ_min_ = −0.24 e Å^−3^

### 

Data collection: *SMART* (Bruker, 2001 ▶); cell refinement: *SAINT-Plus* (Bruker, 2003 ▶); data reduction: *SAINT-Plus*; program(s) used to solve structure: *SHELXS97* (Sheldrick, 2008 ▶); program(s) used to refine structure: *SHELXL97* (Sheldrick, 2008 ▶); molecular graphics: *SHELXTL* (Sheldrick, 2008 ▶); software used to prepare material for publication: *SHELXTL*.

## Supplementary Material

Crystal structure: contains datablocks I, global. DOI: 10.1107/S1600536809004358/pv2132sup1.cif

Structure factors: contains datablocks I. DOI: 10.1107/S1600536809004358/pv2132Isup2.hkl

Additional supplementary materials:  crystallographic information; 3D view; checkCIF report

## Figures and Tables

**Fig. 1.**
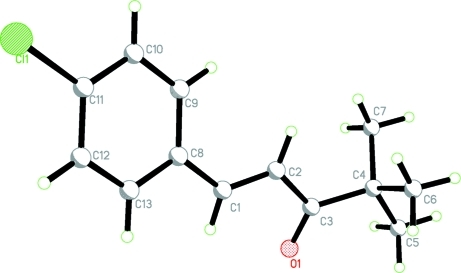
Molecular structure of the title compound showing 30% probability displacement ellipsoids.

**Fig. 2.**
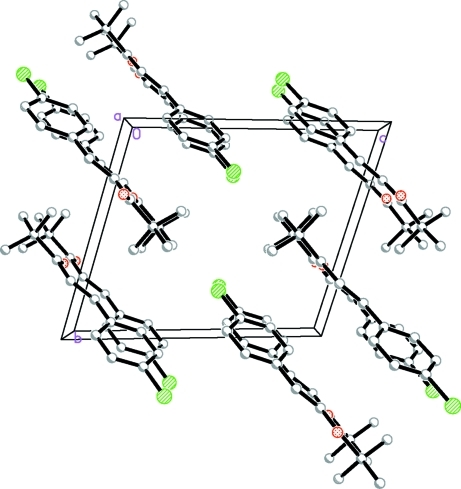
Packing diagram of the title compound.

## supplementary crystallographic information

### Comment

The title compound, 1-(4-chlorophenyl)-4,4-dimethylpentan-3-one, is a very important intermediate in the pesticides industry (Wang *et al.*, 2006). Continuing our work (Xia & Hu 2008) we have now synthsized the title compound, (I). In this article we report the synthesis and crystal structure of (I). Several crystal structures containing phenylprop-2-en-1-one moiety have been recently published, e.g., Anuradha *et al.* (2008); Butcher *et al.* (2007); Gong *et al.* (2008); Harrison *et al.* (2007); Patil *et al.* (2007); Sarojini *et al.* (2007); Thiruvalluvar *et al.* (2007); Thiruvalluvar *et al.* (2008); Xia & Hu (2008).

In the title compound (Fig. 1), the carbonyl and ethenyl groups are not coplanar with the benzene ring; the mean planes of the carbonyl group (atoms O1/C2/C3/C4) and ethenyl group (atoms C2, C3, C4 and C8) are inclined at 35.37 (5) and 36.27 (11) °, respectively, with the mean-plane of the phenyl ring (C8-C13). The bond lengths and bond angles in (I) are in excellent agreement with the corresponding bond lengths and angles reported in the related compounds given above. The molecules are packed in an offset face-to-face arrangement showing π–π stacking interaction involving the benzene rings with centroid-to-centroid distance = 3.586 (4) Å. The structure is devoid of any classical hydrogen bonds (Fig. 2).

### Experimental

Added 3,3-dimethylbutan-2-one (0.0105 mol) to a solution of 4-dichlorobenzaldehyde (0.01 mol) and 60 ml ethanol drop-wise. Then added 0.1 g 50% NaOH solution as catalyst and stirred at 333 K for 4 h (monitored by TLC). A part of solvent was evaporated, then cooled the mixture to 277 K and the precipitate formed were filtered and dried, giving the desired product (yield: 92.1%). Crystals suitable for X-ray structure determination were obtained by slow evaporation of an ethanol solution at room temperature.

### Refinement

The methyl H atom were positioned geometrically (C—H=0.98 Å) and torsion angles refine to fit the electron density [Uĩso~(H) = 1.5U~eq~(C)]. Other H atoms were placed in calculated position (methylene C—H=0.95Å and aromatic C—H=0.95 Å) and refine as riding [Uĩso~(H) = 1.2U~eq~(C)].

### Crystal data

**Table:**

C_13_H_15_ClO	*Z* = 2
*M**_r_* = 222.70	*F*(000) = 236
Triclinic, *P*1	*D*_x_ = 1.266 Mg m^−^^3^
Hall symbol: -P 1	Melting point: 360 K
*a* = 5.6831 (4) Å	Mo *K*α radiation, λ = 0.71073 Å
*b* = 9.9156 (6) Å	Cell parameters from 3394 reflections
*c* = 11.3731 (7) Å	θ = 2.2–27.0°
α = 103.487 (1)°	µ = 0.30 mm^−^^1^
β = 101.160 (1)°	*T* = 173 K
γ = 103.697 (1)°	Block, colorless
*V* = 584.12 (7) Å^3^	0.46 × 0.31 × 0.21 mm

### Data collection

**Table:**

Bruker SMART 1000 CCD diffractometer	2251 independent reflections
Radiation source: fine-focus sealed tube	1989 reflections with *I* > 2σ(*I*)
graphite	*R*_int_ = 0.016
ω scans	θ_max_ = 26.0°, θ_min_ = 1.9°
Absorption correction: multi-scan (*SADABS*; Sheldrick, 2004)	*h* = −6→7
*T*_min_ = 0.875, *T*_max_ = 0.940	*k* = −12→11
4545 measured reflections	*l* = −13→13

### Refinement

**Table:**

Refinement on *F*^2^	Primary atom site location: structure-invariant direct methods
Least-squares matrix: full	Secondary atom site location: difference Fourier map
*R*[*F*^2^ > 2σ(*F*^2^)] = 0.032	Hydrogen site location: inferred from neighbouring sites
*wR*(*F*^2^) = 0.094	H-atom parameters constrained
*S* = 1.07	*w* = 1/[σ^2^(*F*_o_^2^) + (0.0518*P*)^2^ + 0.1538*P*] where *P* = (*F*_o_^2^ + 2*F*_c_^2^)/3
2251 reflections	(Δ/σ)_max_ = 0.001
139 parameters	Δρ_max_ = 0.26 e Å^−^^3^
0 restraints	Δρ_min_ = −0.24 e Å^−^^3^

### Special details

**Table:**

Experimental. ^1^H NMR(400MHz, CDCl3),delta:1.23(s, 9H, 3×CH_3_),7.09(d, J= 1.52Hz, 1H, 2-CH), 7.36(d, J=8.4Hz, 2H, benzene 3,5-H), 7.5(d, J= 8.4Hz, 2H, benzene 2,6-H), 7.60(d,J=15.6Hz, 1H, 1-CH).
Geometry. All e.s.d.'s (except the e.s.d. in the dihedral angle between two l.s. planes) are estimated using the full covariance matrix. The cell e.s.d.'s are taken into account individually in the estimation of e.s.d.'s in distances, angles and torsion angles; correlations between e.s.d.'s in cell parameters are only used when they are defined by crystal symmetry. An approximate (isotropic) treatment of cell e.s.d.'s is used for estimating e.s.d.'s involving l.s. planes.
Refinement. Refinement of *F*^2^ against ALL reflections. The weighted *R*-factor *wR* and goodness of fit *S* are based on *F*^2^, conventional *R*-factors *R* are based on *F*, with *F* set to zero for negative *F*^2^. The threshold expression of *F*^2^ > σ(*F*^2^) is used only for calculating *R*-factors(gt) *etc*. and is not relevant to the choice of reflections for refinement. *R*-factors based on *F*^2^ are statistically about twice as large as those based on *F*, and *R*- factors based on ALL data will be even larger.

### Fractional atomic coordinates and isotropic or equivalent isotropic displacement parameters (Å^2^)

**Table:**

	*x*	*y*	*z*	*U*_iso_*/*U*_eq_	
Cl1	1.79614 (7)	1.22580 (4)	1.45960 (3)	0.03998 (15)	
C1	0.8701 (3)	0.81783 (15)	1.07764 (13)	0.0281 (3)	
H1	0.7164	0.8160	1.0994	0.034*	
C2	0.8553 (3)	0.73017 (15)	0.96684 (13)	0.0292 (3)	
H2	1.0047	0.7274	0.9420	0.035*	
C3	0.6090 (3)	0.63638 (15)	0.88112 (13)	0.0274 (3)	
C4	0.6118 (3)	0.51705 (15)	0.76919 (13)	0.0275 (3)	
C5	0.3442 (3)	0.43184 (18)	0.69238 (15)	0.0415 (4)	
H5A	0.2516	0.3883	0.7452	0.062*	
H5B	0.3480	0.3550	0.6213	0.062*	
H5C	0.2609	0.4974	0.6612	0.062*	
C6	0.7443 (3)	0.41555 (16)	0.81908 (14)	0.0347 (3)	
H6A	0.6574	0.3762	0.8757	0.052*	
H6B	0.9188	0.4701	0.8647	0.052*	
H6C	0.7412	0.3357	0.7487	0.052*	
C7	0.7574 (3)	0.58705 (17)	0.68748 (14)	0.0361 (3)	
H7A	0.7492	0.5121	0.6122	0.054*	
H7B	0.9330	0.6345	0.7350	0.054*	
H7C	0.6827	0.6593	0.6631	0.054*	
C8	1.0991 (3)	0.91697 (14)	1.16967 (13)	0.0273 (3)	
C9	1.3206 (3)	0.96763 (15)	1.13599 (13)	0.0310 (3)	
H9	1.3244	0.9365	1.0510	0.037*	
C10	1.5339 (3)	1.06235 (16)	1.22452 (14)	0.0316 (3)	
H10	1.6825	1.0972	1.2005	0.038*	
C11	1.5282 (3)	1.10569 (14)	1.34851 (13)	0.0291 (3)	
C12	1.3137 (3)	1.05773 (16)	1.38509 (14)	0.0328 (3)	
H12	1.3126	1.0879	1.4706	0.039*	
C13	1.0999 (3)	0.96487 (15)	1.29538 (14)	0.0312 (3)	
H13	0.9506	0.9331	1.3199	0.037*	
O1	0.41421 (19)	0.65539 (12)	0.90060 (10)	0.0373 (3)	

### Atomic displacement parameters (Å^2^)

**Table:**

	*U*^11^	*U*^22^	*U*^33^	*U*^12^	*U*^13^	*U*^23^
Cl1	0.0345 (2)	0.0357 (2)	0.0361 (2)	0.00107 (16)	0.00176 (15)	0.00061 (16)
C1	0.0257 (7)	0.0273 (7)	0.0319 (7)	0.0092 (5)	0.0075 (6)	0.0087 (6)
C2	0.0238 (7)	0.0309 (7)	0.0321 (7)	0.0091 (6)	0.0080 (5)	0.0063 (6)
C3	0.0254 (7)	0.0293 (7)	0.0287 (7)	0.0088 (6)	0.0075 (5)	0.0093 (6)
C4	0.0246 (7)	0.0287 (7)	0.0271 (7)	0.0079 (5)	0.0056 (5)	0.0053 (6)
C5	0.0286 (8)	0.0434 (9)	0.0394 (8)	0.0077 (7)	0.0024 (6)	−0.0039 (7)
C6	0.0376 (8)	0.0324 (8)	0.0378 (8)	0.0137 (6)	0.0122 (6)	0.0121 (6)
C7	0.0434 (9)	0.0384 (8)	0.0333 (8)	0.0166 (7)	0.0163 (7)	0.0134 (6)
C8	0.0289 (7)	0.0237 (7)	0.0299 (7)	0.0106 (6)	0.0065 (5)	0.0071 (5)
C9	0.0318 (8)	0.0323 (7)	0.0277 (7)	0.0087 (6)	0.0085 (6)	0.0068 (6)
C10	0.0272 (7)	0.0324 (7)	0.0342 (8)	0.0067 (6)	0.0087 (6)	0.0097 (6)
C11	0.0283 (7)	0.0219 (6)	0.0320 (7)	0.0065 (5)	0.0024 (6)	0.0039 (5)
C12	0.0360 (8)	0.0310 (7)	0.0284 (7)	0.0095 (6)	0.0081 (6)	0.0039 (6)
C13	0.0286 (7)	0.0312 (7)	0.0333 (7)	0.0081 (6)	0.0112 (6)	0.0067 (6)
O1	0.0247 (5)	0.0442 (6)	0.0382 (6)	0.0109 (5)	0.0093 (4)	0.0016 (5)

### Geometric parameters (Å, °)

**Table:**

Cl1—C11	1.7401 (15)	C6—H6B	0.9800
C1—C2	1.328 (2)	C6—H6C	0.9800
C1—C8	1.464 (2)	C7—H7A	0.9800
C1—H1	0.9500	C7—H7B	0.9800
C2—C3	1.4855 (19)	C7—H7C	0.9800
C2—H2	0.9500	C8—C13	1.396 (2)
C3—O1	1.2185 (17)	C8—C9	1.400 (2)
C3—C4	1.5281 (19)	C9—C10	1.383 (2)
C4—C5	1.5242 (19)	C9—H9	0.9500
C4—C7	1.535 (2)	C10—C11	1.384 (2)
C4—C6	1.5368 (19)	C10—H10	0.9500
C5—H5A	0.9800	C11—C12	1.378 (2)
C5—H5B	0.9800	C12—C13	1.385 (2)
C5—H5C	0.9800	C12—H12	0.9500
C6—H6A	0.9800	C13—H13	0.9500
			
C2—C1—C8	126.64 (13)	H6B—C6—H6C	109.5
C2—C1—H1	116.7	C4—C7—H7A	109.5
C8—C1—H1	116.7	C4—C7—H7B	109.5
C1—C2—C3	121.13 (13)	H7A—C7—H7B	109.5
C1—C2—H2	119.4	C4—C7—H7C	109.5
C3—C2—H2	119.4	H7A—C7—H7C	109.5
O1—C3—C2	120.51 (13)	H7B—C7—H7C	109.5
O1—C3—C4	122.12 (13)	C13—C8—C9	118.03 (13)
C2—C3—C4	117.37 (12)	C13—C8—C1	119.85 (13)
C5—C4—C3	110.10 (12)	C9—C8—C1	122.13 (13)
C5—C4—C7	109.81 (12)	C10—C9—C8	121.03 (13)
C3—C4—C7	108.91 (11)	C10—C9—H9	119.5
C5—C4—C6	110.00 (12)	C8—C9—H9	119.5
C3—C4—C6	108.29 (11)	C9—C10—C11	119.20 (13)
C7—C4—C6	109.71 (12)	C9—C10—H10	120.4
C4—C5—H5A	109.5	C11—C10—H10	120.4
C4—C5—H5B	109.5	C12—C11—C10	121.34 (13)
H5A—C5—H5B	109.5	C12—C11—Cl1	119.52 (11)
C4—C5—H5C	109.5	C10—C11—Cl1	119.13 (11)
H5A—C5—H5C	109.5	C11—C12—C13	119.01 (13)
H5B—C5—H5C	109.5	C11—C12—H12	120.5
C4—C6—H6A	109.5	C13—C12—H12	120.5
C4—C6—H6B	109.5	C12—C13—C8	121.37 (13)
H6A—C6—H6B	109.5	C12—C13—H13	119.3
C4—C6—H6C	109.5	C8—C13—H13	119.3
H6A—C6—H6C	109.5		
			
C8—C1—C2—C3	−178.96 (13)	C13—C8—C9—C10	0.0 (2)
C1—C2—C3—O1	12.6 (2)	C1—C8—C9—C10	179.39 (13)
C1—C2—C3—C4	−167.46 (13)	C8—C9—C10—C11	0.9 (2)
O1—C3—C4—C5	−0.29 (19)	C9—C10—C11—C12	−0.8 (2)
C2—C3—C4—C5	179.76 (12)	C9—C10—C11—Cl1	−179.58 (11)
O1—C3—C4—C7	120.17 (15)	C10—C11—C12—C13	−0.3 (2)
C2—C3—C4—C7	−59.79 (16)	Cl1—C11—C12—C13	178.49 (11)
O1—C3—C4—C6	−120.59 (15)	C11—C12—C13—C8	1.3 (2)
C2—C3—C4—C6	59.46 (16)	C9—C8—C13—C12	−1.1 (2)
C2—C1—C8—C13	−158.39 (14)	C1—C8—C13—C12	179.47 (13)
C2—C1—C8—C9	22.2 (2)		

## References

[bb1] Anuradha, N., Thiruvalluvar, A., Mahalinga, M. & Butcher, R. J. (2008). *Acta Cryst.* E**64**, o2118–o2119. 10.1107/S1600536808032807 PMC2959522 21580981

[bb2] Bruker (2001). *SMART* Bruker AXS Inc., Madison, Wisconsin, USA.

[bb3] Bruker (2003). *SAINT-Plus* Bruker AXS Inc., Madison, Wisconsin, USA.

[bb4] Butcher, R. J., Yathirajan, H. S., Narayana, B., Mithun, A. & Sarojini, B. K. (2007). *Acta Cryst.* E**63**, o30–o32.

[bb5] Gong, Z.-Q., Liu, G.-S. & Xia, H.-Y. (2008). *Acta Cryst.* E**64**, o151. 10.1107/S1600536807063489 PMC2915219 21200716

[bb6] Harrison, W. T. A., Ravindra, H. J., Kumar, M. R. S. & Dharmaprakash, S. M. (2007). *Acta Cryst.* E**63**, o4675.

[bb7] Patil, P. S., Chantrapromma, S., Fun, H.-K. & Dharmaprakash, S. M. (2007). *Acta Cryst.* E**63**, o1738–o1740.

[bb8] Sarojini, B. K., Yathirajan, H. S., Mustafa, K., Sarfraz, H. & Bolte, M. (2007). *Acta Cryst.* E**63**, o4448.

[bb9] Sheldrick, G. M. (2004). *SADABS* University of Göttingen, Germany.

[bb10] Sheldrick, G. M. (2008). *Acta Cryst.* A**64**, 112–122. 10.1107/S0108767307043930 18156677

[bb11] Thiruvalluvar, A., Subramanyam, M., Butcher, R. J., Adhikari, A. V. & Karabasanagouda, T. (2007). *Acta Cryst.* E**63**, o4716. 10.1107/S1600536808017200 PMC2961704 21202897

[bb12] Thiruvalluvar, A., Subramanyam, M., Butcher, R. J., Karabasanagouda, T. & Adhikari, A. V. (2008). *Acta Cryst.* E**64**, o1263. 10.1107/S1600536808017200 PMC2961704 21202897

[bb13] Wang, Y., Hu, A.-X., Chen, P., Chen, M. & Liu, Zh.-L. (2006). *Chin. Agrochem.* **45**, 397–398.

[bb14] Xia, L. & Hu, A.-X. (2008). *Acta Cryst.* E**64**, o1983. 10.1107/S1600536808029838 PMC2959386 21201183

